# Identification of Chromosomes and Chromosome Rearrangements in Crop Brassicas and *Raphanus sativus*: A Cytogenetic Toolkit Using Synthesized Massive Oligonucleotide Libraries

**DOI:** 10.3389/fpls.2020.598039

**Published:** 2020-12-23

**Authors:** Neha Agrawal, Mehak Gupta, Surinder S. Banga, JS (Pat) Heslop-Harrison

**Affiliations:** ^1^Department of Plant Breeding and Genetics, Punjab Agricultural University, Ludhiana, India; ^2^Department of Genetics and Genome Biology, University of Leicester, Leicester, United Kingdom; ^3^South China Botanical Garden, Chinese Academy of Sciences, Guangzhou, China

**Keywords:** Oligo-FISH, chromosomes, translocations, *Brassica*, karyotypes, oligonucleotides, evolution, genomics

## Abstract

Crop brassicas include three diploid [*Brassica rapa* (AA; 2*n* = 2*x* = 16), *B. nigra* (BB; 2*n* = 2*x* = 18), and *B. oleracea* (CC; 2*n* = 2*x* = 20)] and three derived allotetraploid species. It is difficult to distinguish *Brassica* chromosomes as they are small and morphologically similar. We aimed to develop a genome-sequence based cytogenetic toolkit for reproducible identification of *Brassica* chromosomes and their structural variations. A bioinformatic pipeline was used to extract repeat-free sequences from the whole genome assembly of *B. rapa*. Identified sequences were subsequently used to develop four c. 47-mer oligonucleotide libraries comprising 27,100, 11,084, 9,291, and 16,312 oligonucleotides. We selected these oligonucleotides after removing repeats from 18 identified sites (500–1,000 kb) with 1,997–5,420 oligonucleotides localized at each site in *B. rapa*. For one set of probes, a new method for amplification or immortalization of the library is described. oligonucleotide probes produced specific and reproducible *in situ* hybridization patterns for all chromosomes belonging to A, B, C, and R (*Raphanus sativu*s) genomes. The probes were able to identify structural changes between the genomes, including translocations, fusions, and deletions. Furthermore, the probes were able to identify a structural translocation between a pak choi and turnip cultivar of *B. rapa.* Overall, the comparative chromosomal mapping helps understand the role of chromosome structural changes during genome evolution and speciation in the family Brassicaceae. The probes can also be used to identify chromosomes in aneuploids such as addition lines used for gene mapping, and to track transfer of chromosomes in hybridization and breeding programs.

## Introduction

The genus *Brassica* (family Brassicaceae, with some 37 species) includes six major vegetable or oil crops: three diploid [*B. rapa* (AA genome composition, 2*n* = 20), *B. nigra* (BB, 2*n* = 16), and *B. oleracea* (CC, 2*n* = 18)] and three allotetraploid species [*B. juncea* (AABB, 2*n* = 36), *B. napus* (AACC, 2*n* = 38), and *B. carinata* (BBCC, 2*n* = 34)]. The allotetraploid brassicas evolved from pair-wise natural hybridizations between the three basic diploids. Although monophyletic, evolution of diploid *Brassica* genomes (A, B, and C) is complex (Lagercrantz and Lydiate, 1996; Lagercrantz, 1998). Inferences from comparative genome biology and phylogenetic reconstructions from whole genome sequences of brassica diploids are consistent with their common origin from an ancient paleohexaploid (γ event), followed by two whole-genome duplications (Wang et al., 2011; Zhang et al., 2018; Sun et al., 2019) and an additional whole-genome triplication (WGT), leaving extant brassica genomes as massively rearranged versions of an ancestral paleo-hexaploid genome. The chromosome structural changes resulted from chromosome breakages, fusions, inversions, and deletions after each cycle of polyploidy and diploidization (Schranz et al., 2006; Mandakova and Lysak, 2008; Schubert and Lysak, 2011). Despite the erosion of collinearity, high synteny and DNA sequence homologies continue to exist among Brassicaceae genomes (Tang et al., 2008; Cheng et al., 2012), representing regions with more conserved gene order or synteny blocks. There are 24 (A–X) conserved genome blocks (GBs) or ancestral karyotypes (AK) in the family Brassicaceae (Schranz et al., 2006). Each *Brassica* genome has three or six regions that are orthologous to *Arabidopsis thaliana* (Lysak et al., 2005, 2007; Cheng et al., 2014). These also harbor highly repeated sequences and complicated centromeric regions relative to *A. thaliana* (Lagercrantz and Lydiate, 1996; Lagercrantz, 1998; Lan et al., 2000; Chalhoub et al., 2014; Liu et al., 2014; Yang et al., 2016; Zhang et al., 2018). Though gene content evolution mirrored genome changes (Cheng et al., 2012; Tang et al., 2012), orthologs in the syntenic regions retained their functionality. Knowledge of syntenic genes and genomic regions among closely related species is important to explain genome diversification (Lyons et al., 2008). Furthermore, genetic exchanges in the regions of shared synteny are vital for mobilizing genes of interest across species domains, without precipitating non-compensating translocations. *In silico* analysis of DNA sequence data has been vital for the understanding of evolutionary mechanisms that framed structure of existing plant genomes (Salse and Feuillet, 2011).

Fluorescent *in situ* hybridization (FISH) is a powerful molecular cytogenetic technique to characterize karyotype variation at chromosome level by direct localization of repetitive DNA sequences on plant chromosomes (Jiang and Gill, 2006; Patokar et al., 2016; Song et al., 2020), enabling identification of chromosomes even in species with small and morphologically indistinguishable chromosomes, and comparison of chromosomal organization between species. However, such probes can be inconsistent in chromosome identification for multiplicity of repeat sequences and variations in their genomic locations (Mukai et al., 1993; Fransz et al., 1998; Kato et al., 2004; Danilova et al., 2012; Komuro et al., 2013; Koo et al., 2016; Amosova et al., 2017; Krivankova et al., 2017; Hou et al., 2018; Said et al., 2018), and repetitive DNA sequences with suitable genomic locations may not exist. BAC-based chromosome painting techniques have been used to construct high-resolution karyotypes (Kulikova et al., 2001; Pecinka et al., 2004; Zhang et al., 2006; Xiong and Pires, 2011; Wang et al., 2012) and identify chromosome structural variations (Lysak et al., 2005, 2006; Mandakova and Lysak, 2008; Idziak et al., 2011, 2014; Peters et al., 2012; Szinay et al., 2012; Mandakova et al., 2013). In a remarkable experiment, Lysak et al. (2006) explained the origin of each chromosome of *A. thaliana* relative to the ancestral *n* = 8 karyotype. These involved four chromosomal inversions, two translocations and three chromosome fusion events based on ordered BAC pools. Mandakova and Lysak (2008) also used multiple selected BACs as probes to explain monophyletic origin of the *x* = 7 tribes in Brassicaceae family through reduction of chromosome number from *n* = 8 in ancestral karyotype to *n* = 7, with different fusion and intrachromosomal inversion events. Mandakova et al. (2019) combined BAC-based chromosome painting, genomic *in situ* hybridization (GISH) and multi-gene phylogenetics to explain the role of post polyploidy chromosome structural variation in the origin and evolution of the *Camelina sativa* polyploid complex. However, identifying genetically mapped BACs with complete genome coverage is a challenge in most species; repetitive DNA sequences in the target DNA and BAC probes can cause non-specific hybridization. Development of repeat-free probes has proved difficult in some species (Bertioli et al., 2013), although in some cases repetitive DNA, particularly derived from repetitive DNA, may be valuable to identify different genomes in hybrids (Santos et al., 2015; Huang et al., 2020).

Use of massive pools of short synthetic oligonucleotides as probes for chromosomal *in situ* hybridization can allow design of probes to label any part of a chromosome as a band, or be designed to label (“paint”) a complete chromosome (Beliveau et al., 2015; Braz et al., 2018; Šimoníková et al., 2019). The oligonucleotide libraries use a defined set of unique sequences, selected *in silico* out of assembled genome sequences with chromosomal region specificity. These are highly sensitive and provide consistent chromosome labeling and signal intensity. Synthesis and labeling of massive oligonucleotide pools typically require thousands of oligonucleotides, 20–100 bp long. Their synthesis is now possible with a range of newly available commercial sources (Affymetrix, Combimatrix, Twist, inkjet printing/Agilent, and Mycroarray/Arbor Biosciences), using whole genome draft assemblies to anchor genome sequence information directly to chromosome topographies for getting a phylogenetic view of species. We expect this approach to provide a correct view of evolutionary relationships among species as single copy genomic regions are used to develop oligonucleotide pools. oligonucleotide libraries have been used as robust FISH probes in many plant species to construct molecular cytogenetic karyotypes (Braz et al., 2018; Meng et al., 2018), characterize chromosomal rearrangements and visualize homoeologous pairing among related species (Han et al., 2015; Qu et al., 2017; Xin et al., 2018) and integrate pseudomolecules of reference genome sequence of *Musa acuminata* spp. *malaccensis* “DH Pahang” to individual chromosomes *in situ* (Šimoníková et al., 2019).

Here, we aimed to develop an easy, robust and efficient oligonucleotide based cytogenetic toolkit for consistent and reproducible characterization of chromosomes or their structural variants in *Brassica*. Karyotype construction in Brassicaceae family has been challenging because of small chromosome size and the absence of cytological landmarks. The probes are useful for determining chromosome evolution or homology in the family Brassicaceae and applicable in genomic studies and in plant breeding aiming to exploit the germplasm pool. These probes allow comparative studies using oligonucleotides from conserved DNA sequences from one species in other genetic related species. We constructed four oligonucleotide libraries from 18 identified regions of *B. rapa* assembled genome sequence and tested them to identify all chromosomes of A, B, C, and R (*Raphanus sativus*) genomes. Our probes could also differentiate chromosome arms and pre-existing translocations in a commercial genotype of *B. rapa*. We also report an improved method for immortalization of oligonucleotide libraries to optimize the cost of oligonucleotide paints, which can otherwise be expensive or require demanding optimization.

## Materials and Methods

### Plant Materials

*Brassica rapa, B. nigra, B. oleracea*, and *Raphanus sativus* were used for comparative FISH analysis. Seeds of *B. rapa* ssp. *chinensis* cv. Chiifu-401 (pak choi or Chinese cabbage) were obtained from the University of Warwick, United Kingdom and *B. rapa* ssp. *rapa* cv. Turnip Purple Top Milan was sourced from Mr. Fothergill’s Seeds, United Kingdom. Other seeds of *B. nigra, B. oleracea*, and *R. sativus* were from the germplasm collections maintained at Punjab Agricultural University, Ludhiana, India.

### Design of Oligonucleotide Pools

**FIGURE 1:**
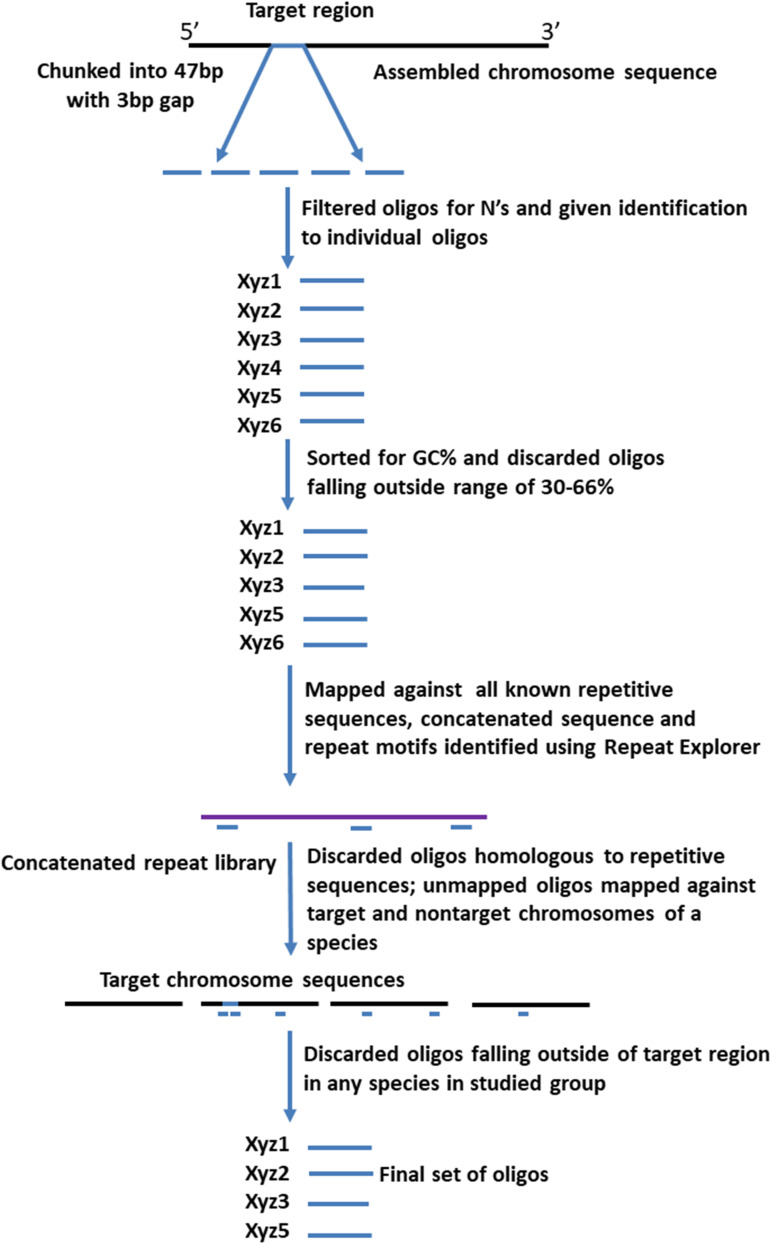
Pipeline for designing of synthetic oligonucleotide libraries (Table 1) to chromosome loci. After generating 47 bp oligonucleotides (“oligos”) from the target regions, those with ambiguities or extreme GC-content, or that hybridize (assessed by mapping *in silico)* to repetitive sequences and non-target chromosomes, are discarded. The final pool has oligonucleotides hybridizing specifically to the target chromosomal regions.

**FIGURE 2:**
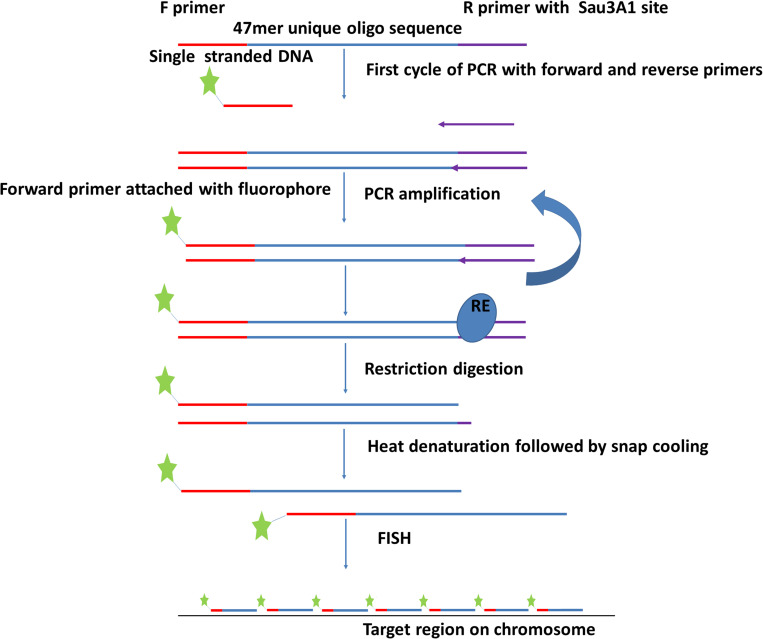
Flowchart depicting procedure for amplification of oligonucleotide libraries (immortalization). The oligonucleotides (Figure 1) are synthesized with 5′ and 3′ primers and amplified by PCR using one labeled and one unlabeled primer. After restriction enzyme digestion to remove the unlabeled end complementary to the reverse primer, the labeled product is hybridized to chromosome preparations.

**FIGURE 3:**
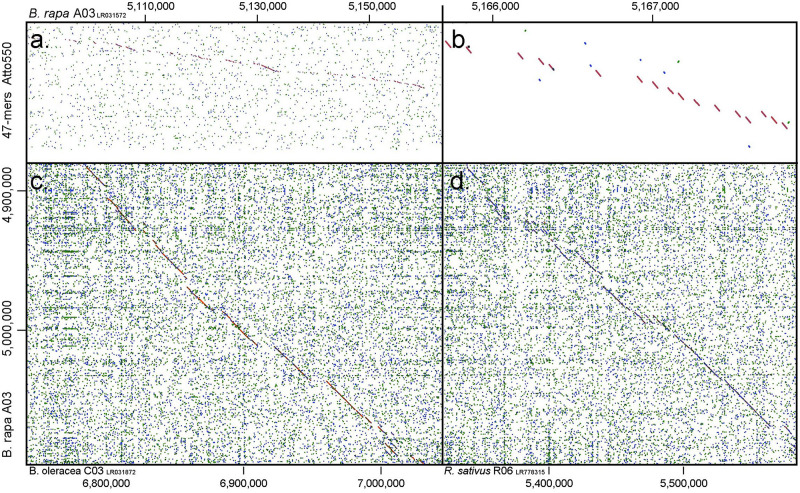
Dot plots depicting similarity between **(a,b)** oligonucleotide pool of 47-mers labeled with Atto550 and *B. rapa* chromosome A03 (1.0 gramene.org, y-axis): gaps show regions where nucleotides have been deleted by the selection procedure; and comparing A03 with **(c)** *B. oleracea* C03 and **(d)** *Raphanus sativus* R06. Diagonal lines show regions of high similarity where oligonucleotide probes would hybridize to both genomes, while gaps indicate insertions in one of the genomes or regions of lower homology.

Four different sets of oligonucleotide pools were designed, each one labeled with a different fluorophore to create a multi-color barcode for identification of individual *B. rapa* chromosomes (Supplementary Table 1). One to three ranges of 0.5–1 MB in size were selected from different regions (sub-telomeric, intercalary, or sub-centromeric) of the DNA sequences of all of the 10 chromosomes of *B. rapa* (2*n* = 2*x* = 20) to create unique chromosome specific hybridization patterns upon simultaneous hybridization with four oligonucleotide sets. We downloaded chromosome assemblies of *B. rapa* cv. Chiifu-401 (synonyms Chiffu and Chifu) V_2.5, *B. oleracea* V_1, *Raphanus sativus* V_1 from the public *Brassica* database (BRAD^1^). The *B. nigra* chromosome assembly was kindly provided by Isobel Parkin (Saskatoon; Perumal et al., 2020). Linux command lines were used to split the target regions for probe design into 47 bp fragments with a 3 bp gap to prevent potential steric interference by adjacent oligonucleotide probes during *in situ* hybridization. We retained fragments with 30–66% GC content. oligonucleotides were further screened in sequential steps against all known repetitive sequences, including rDNA, chloroplast, published repeats (pBo, pBc families; Harrison and Heslop-Harrison, 1995) and a new repetitive motif library developed from unassembled, raw Illumina reads. Frequencies of all 32-mers (k-mer) were calculated, and the most abundant 5,000 were concatenated. Any 47-mer oligonucleotides mapping to approximately 28 of more bases of the concatenated sequence were discarded. We also tested against highly repetitive motifs from graph-based clustering of the raw reads using RepeatExplorer (Novak et al., 2013) to remove further repeats. The depleted libraries were then mapped back to published whole genome sequences (Bowtie2). Firstly, in *B. rapa*, any oligonucleotides mapping outside the target region were discarded (Figure 1) and primers added for PCR amplification for some pools (Figure 2). Reads from the target region were then mapped to reference with *B. nigra* chromosome to select sequence(s) common between the species occurring only in homoeologous and syntenic genome regions (Figure 3). Final oligonucleotides sets were also mapped to *B. oleracea* and *R. sativus* and in some cases screened against inclusion of repetitive sequences from these species that were less abundant in the source *B. rapa* genome. *In silico* hybridization simulations showed characteristic binding patterns of four oligonucleotide libraries to the genomes A, B, C, and R. Dot-plots were constructed among various chromosomes belonging to different genomes around the regions where probes were hybridizing to estimate degree of similarity and identify any rearrangements or major sequence insertions between the species (Figure 3).

### Immortalization of Oligonucleotide Libraries

Oligonucleotides were designed with addition of a 20 bp 5′ primer annealing site (complementary to T7 primer), 47 bp of the unique oligo, and a 20 nt 3′ primer annealing site containing *Sau*3AI restriction site (GATCTCTGCATCTAGTAATG) (Figure 2). Unlabeled oligo libraries were ordered from Arbor Biosciences (Ann Arbor, MI). Each synthesized library contained 100 ng of DNA. These libraries were amplified and labeled simultaneously using PCR. Briefly, the PCR mixture of 50 μl reaction included 1 pmol DNA from the oligo library pool, 25 μM each of F (T7 primer 5′end labeled with the fluorochrome Cy5) and R (CTAGAAGTTACTGAGAGATC) primers, (underlined sequence depicts *Sau*3AI restriction site), 40 mM dNTPs, 1 unit of Platinum SuperFi DNA Polymerase in 5X high fidelity (HF) buffer and enhancer. The reactions were cycled as: 98°C for 30 s, 2x (98°C for 30 s, 59°C for 10 s, and 72°C for 10 s), continuing with a 2°C reduction each cycle till 53°C, 15–20x (98°C for 10 s, 53°C for 10 s, and 72°C for 10 s), 72°C for 1 min then held at 15°C. After amplification, the PCR product was digested with *Sau*3AI (Figure 2) to remove 3′ primers. Digested product was then purified using commercially available cycle purification kit of DNA from Omega and used as a probe for *in situ* hybridization.

### Chromosome Preparations and *in situ* Hybridization Protocol

Metaphase chromosome preparations and *in situ* hybridization was performed according to Schwarzacher and Heslop-Harrison (2000) with minor modifications. The most stringent post-hybridization washes were carried out in 0.1X SSC at 42°C. The custom synthesized and labeled oligonucleotide library pools were directly used as FISH probes. Preparations were counterstained with DAPI in VectaShield antifade solution. The slides were examined and FISH images were captured using a Nikon Eclipse N80i fluorescent microscope equipped with a DS-QiMc monochromatic camera (Nikon, Japan). Raw images were processed with Adobe Photoshop using only functions that affect the whole image equally.

## Results

### Development of Oligonucleotide-Based Probes for Chromosome Identification in *Brassica*

**FIGURE 4:**
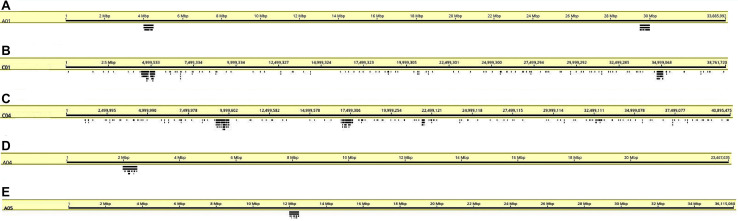
Coverage graphs (stacked bars below chromosomes) depicting hybridization simulations of oligonucleotide libraries made from the *B. rapa* A genome on chromosomes **(A)** A01, **(B)** C01, **(C)** C04, **(D)** A04, and **(E)** A05. Sites are only within target regions on the A genome chromosomes as expected from the design strategy, but a few hybridization sites are seen elsewhere on C genome chromosomes **(B,C)**.

We designed oligonucleotides from 18 different regions of *B. rapa* genome (BRAD *B. rapa* Version 2.5) using the strategy outlined in Figure 1. Genome coordinates for 18 identified regions are available in table 1. Each chromosomal region comprised 1,997–5,420 oligonucleotides, spanning over 500–1,000 kb (Table 1). Three libraries were custom synthesized and labeled with dyes Atto488-Green, Atto550-Red and Atto594-Yellow for use as robust probes. We also amplified an unlabeled library and labeled it with Cy5-Far Red, using standard PCR techniques (without an RNA intermediate) to develop an immortal probe. We retained 63,787 oligonucleotides (47 bp long) in four libraries (27,100, 11,084, 9,291, and 16,312 oligonucleotides, chromosomal locations and sequences shown in Supplementary Table 1). Simulated hybridization of oligonucleotides with whole genome sequence of *B. rapa* produced results in form of 18 intense peaks on ten chromosomes of *B. rapa*. The designed oligonucleotide-pools generated 18 distinct FISH signals on 10 chromosomes of *B. rapa* (chromosomes A01–A10) and the characteristic hybridization patterns identified all individual *B. rapa* chromosomes. Importantly, all the regions used for probe design gave signals. We also tested transferability of A genome probes to other brassica and radish genomes (Figures 3a–d). Simulations had earlier shown that the same oligonucleotide sets could depict 16, 18, and 18 sites in B (*B. nigra*), C (*B. oleracea*), and R (*R. sativus*) genomes respectively. The position (along the chromosome between centromere and telomere), intensity (number of probes with high homology) and width (region showing many probes hybridizing) of the respective peaks differed from those observed for A-genome. As an example, chromosome C01 showed the same two peaks as recorded in A01, in the same order between telomere and centromere (Figures 4A,B). The first peak, observed by hybridization of probes, complemented a region 4 Mbp from the start of chromosome A01 and 4.8 Mbp from start of C01. Hybridization of the probes produced the second peak on A01, with a region initializing at 29.5 Mbp from the start; C01 produced this peak at 34.5 Mbp. The number of probes predicted to hybridize

**TABLE 1:** Details of design of synthetic massive oligonucleotide pools at each chromosomal locus (A, B, and C genomes and chromosomes number), with start and end position along the sequence and this span, the number of oligonucleotides (oligos) designed, density of oligonucleotides over the region, and fluorochrome label used.

**Label**	**Start position (Mb)**	**End position (Mb)**	**Number of oligos**	**Chromosome**	**Origin**	**Span (kb)**	**Oligo density/kb**
Red (Atto550)	5	6	1,997	A03	A03	1000	2.0
Red (Atto550)	34.9	36	2,253	A05	A05	1010	2.2
Red (Atto550)	20	21	3,858	A06	A06	1000	3.9
Red (Atto550)	10	11	2,976	A09	A09	1000	3.0
Red (Atto550)	37.8	38.3	2,245	B01	A05	500	4.5
Red (Atto550)	10.8	11.6	1,287	B04	A09	800	1.6
Red (Atto550)	17.3	18.3	1,361	B04	A06	1000	1.4
Red (Atto550)	4.5	6.1	2,000	B08	A03	1600	1.3
Red (Atto550)	4.7	6.2	1,530	C03	A03	1500	1.0
Red (Atto550)	14.2	15.5	2,393	C05	A06	1300	1.8
Red (Atto550)	33.9	34.4	653	C09	A09	500	1.3
Red (Atto550)	2.1	3.5	1,003	R03	A03	1400	0.7
Red (Atto550)	36.9	38.1	1,294	R05	A05	1200	1.1
Red (Atto550)	16.7	17.3	1,006	R06	A06	600	1.7
Red (Atto550)	5.1	5.8	1,063	R09	A09	700	1.5
Green (Atto488)	4	4.5	5,420	A01-1	A01-1	500	10.8
Green (Atto488)	29.5	30	4,615	A01-2	A01-2	500	9.2
Green (Atto488)	2	2.5	4,512	A04	A04	500	9.0
Green (Atto488)	12	12.5	3,906	A05	A05	500	7.8
Green (Atto488)	4	4.5	4,794	A09-1	A09-1	500	9.6
Green (Atto488)	40	40.5	3,853	A09-2	A09-2	500	7.7
Green (Atto488)	4	4.45	2,023	B05	A01-1	450	4.5
Green (Atto488)	17.5	18	1,057	B06-1	A05	500	2.1
Green (Atto488)	33.9	34.2	1,076	B06-2	A04	300	3.6
Green (Atto488)	2.9	3.3	1,225	B07-1	A01-2	400	3.1
Green (Atto488)	10.3	10.9	1,453	B07-2	A09-1	600	2.4
Green (Atto488)	44.6	45.25	1,060	B08	A09-2	600	1.8
Green (Atto488)	4.4	5.2	4,020	C01-1	A01-1	800	5.0
Green (Atto488)	34.7	35	1,759	C01-2	A01-1	300	5.9
Green (Atto488)	9.2	10	2,119	C04-1	A04	800	2.6
Green (Atto488)	16.9	17.6	1,844	C04-2	A05	700	2.6
Green (Atto488)	21.9	22	583	C04-3	A04	100	5.8
Green (Atto488)	26.7	27.3	2,667	C08	A09-2	600	4.4
Green (Atto488)	3.75	4	1,513	C09	A09-1	250	6.1
Green (Atto488)	4.5	5	973	R01	A05	500	1.9
Green (Atto488)	11.7	12.1	954	R01	A09-2	400	2.4
Green (Atto488)	45.3	46	1,167	R01	A01-2	700	1.7
Green (Atto488)	1.5	1.9	1,646	R02	A01-1	400	4.1
Green (Atto488)	46.7	47.5	940	R04	A04	800	1.2
Green (Atto488)	4.7	5.1	1,030	R09	A09-1	400	2.6
Yellow (Atto594)	34	35	2,291	A06	A06	1000	2.3
Yellow (Atto594)	25	26	2,495	A07	A07	1000	2.5
Yellow (Atto594)	4	5	2,225	A10-1	A10-1	1000	2.2
Yellow (Atto594)	15	16	2,280	A10-2	A10-2	1000	2.3
Yellow (Atto594)	31.8	32.9	2,271	B02-1	A10-2	1100	2.1
Yellow (Atto594)	41.1	41.8	2,281	B02-2	A06	700	3.3
Yellow (Atto594)	24.5	26	2,478	B03	A07	1500	1.7
Yellow (Atto594)	4.3	5.2	1,219	C06-1	A07	900	1.4
Yellow (Atto594)	38.3	39.2	1,233	C06-2	A10-1	900	1.4
Yellow (Atto594)	33.5	34.9	1,827	C07	A06	1400	1.3
Yellow (Atto594)	36	37.5	1,840	C09	A10-2	1500	1.2
Yellow (Atto594)	9	10.4	1,232	R04	A06	1400	0.9
Yellow (Atto594)	19.1	20.2	1,210	R07	A10-2	1100	1.1
Yellow (Atto594)	28.3	29.6	1,320	R09	A07	1300	1.0
Far Red (Cy5)	5	6	4,897	A02-1	A02-1	1000	4.9
Far Red (Cy5)	25	26	3,489	A02-2	A02-2	1000	3.5
Far Red (Cy5)	25	26	2,494	A07	A07	1000	2.5
Far Red (Cy5)	2	3	2,473	A08-1	A08-1	1000	2.5
Far Red (Cy5)	12	13	2,959	A08-2	A08-2	1000	3.0
Far Red (Cy5)	24.3	26	2,486	B03	A07	1700	1.5
Far Red (Cy5)	3	3.5	1,172	B04	A02-2	500	2.3
Far Red (Cy5)	21.5	23.2	1,334	B05	A02-1	1700	0.8
Far Red (Cy5)	20.9	22	1,183	B07	A08-2	1100	1.1
Far Red (Cy5)	6.8	7.9	3,295	C02-1	A02-1	1100	3.0
Far Red (Cy5)	38.7	40.3	2,214	C02-2	A02-2	1600	1.4
Far Red (Cy5)	4.3	5.2	1,218	C06-1	A07	900	1.4
Far Red (Cy5)	6.2	7.5	1,781	C06-2	A08-2	1300	1.4
Far Red (Cy5)	0.3	1.1	656	C08	A08-1	800	0.8
Far Red (Cy5)	34.8	35.7	986	R01	A02-2	900	1.1
Far Red (Cy5)	15.7	17	1,250	R02	A02-1	1300	1.0
Far Red (Cy5)	11.9	12.8	909	R08	A08-1	900	1.0
Far Red (Cy5)	28.3	29.6	1,319	R09	A07	1300	1.0

**FIGURE 5:**
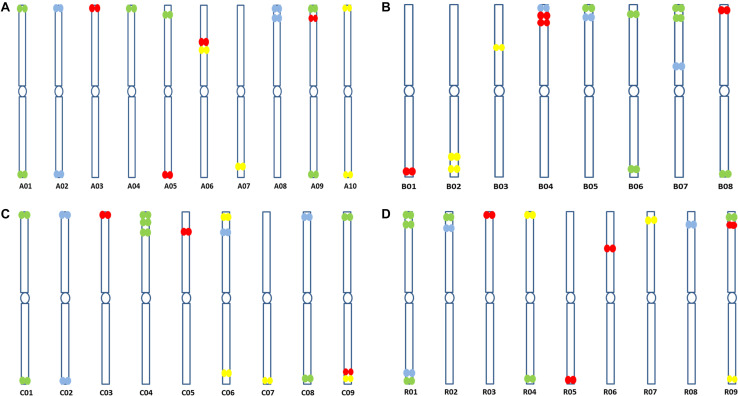
Predicted ideotypes of **(A)** *B. rapa* (A genome, 2*n* = 2*x* = 20) **(B)** *B. nigra* (B genome, 2*n* = 2*x* = 16), **(C)** *B. oleracea* (C genome, 2*n* = 2*x* = 18), and **(D)** *R. sativus* (R genome, 2*n* = 2*x* = 18) showing sites of hybridization of the four oligonucleotide libraries (red, yellow, green, and cyan) on diagrammatic chromosomes.

**FIGURE 6:**
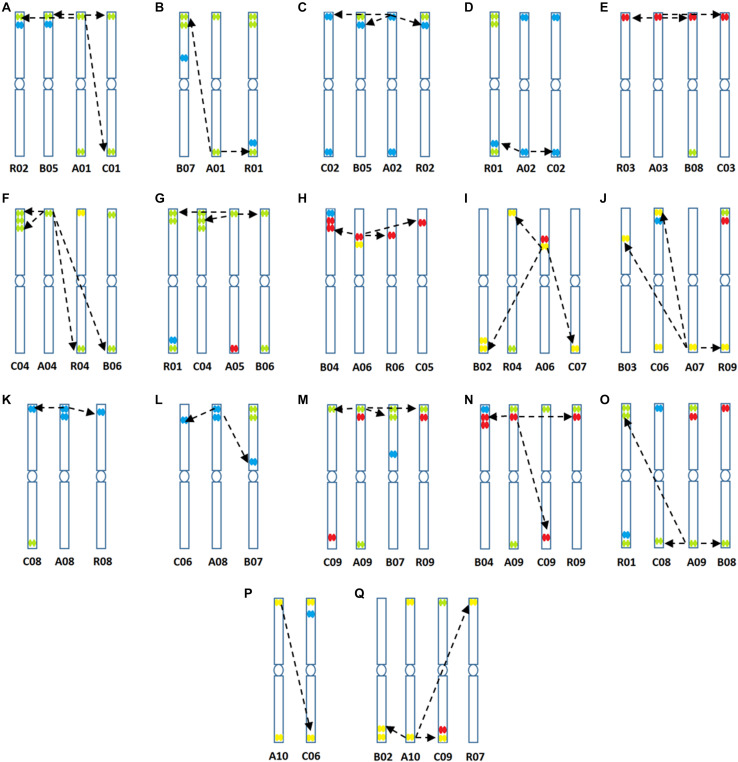
**(A–Q)** Predicted structural chromosomal rearrangements among cultivated genomes of *Brassica* based on sequence data and locations of hybridization of the four oligonucleotide libraries.

*The full set of sequences is given in Supplementary Table 1.*

at the peaks in C01 was lower than those observed in A01 by around a factor of two, presumably because of divergence of the low-copy sequences in the region. Chromosome C01 is slightly longer than A01 (by about 5 Mbp). The first C01 signal peak was wider than the A01 equivalent, suggesting multiple insertions in C01 regarding A01. However, the second C01 peak was narrower, indicating sequence expansion of A01 relative to C01. C04 peaks shared some similarities with peaks of both A04 and A05, although the relationship between these chromosomes was not as strong as observed between A01 and C01 (Figures 4C–E). In both A04 and C04 the first peak hybridized with the same oligonucleotides, although the hybridization occurred in C04 further into the chromosome by approximately 7.4 Mbp. The sequences on the A05 peak were also present in C04, in a different location and in reverse orientation. Both C04 peaks had low signal intensity, showing divergence. The homoeologous chromosomes have evolved through insertions, deletions and translocations (and also through repetitive sequence homogenization, although this evolutionary mechanism would not be detected by the low-copy oligonucleotides). A dot-plot shows the locations of the 47 bp oligonucleotides on the chromosome-of-origin for a section of *B. rapa* chromosome A03 (Figures 3a,b) with gaps showing oligonucleotides that were deleted by the selection procedure in Figure 1. Additional dot-plot analyses show the comparison of the A03 chromosomes, with insertions (gaps) and regions of weaker and strong homology with (diagonal lines) (Figures 3c,d) with *B. oleracea* C03 and *Raphanus sativus* R06. Differences in the location, size and intensities of the peak signals allowed identification of all chromosomes of A, B, C, and R genomes (Figures 5A–D). *In silico* developed ideotypes also revealed chromosomal rearrangements such as translocations and fusion events for genome coordinates used for probe synthesis (Figures 6A–Q). As allotetraploid *Brassica* species evolved from the direct pairwise hybridizations between three diploid species, so the synthesized probes can be efficiently used to identify all chromosomes of *B. juncea, B. carinata*, and *B. napus*.

### Comparative *in situ* Studies Using Developed Oligonucleotide-Based FISH Probes

**FIGURE 7:**
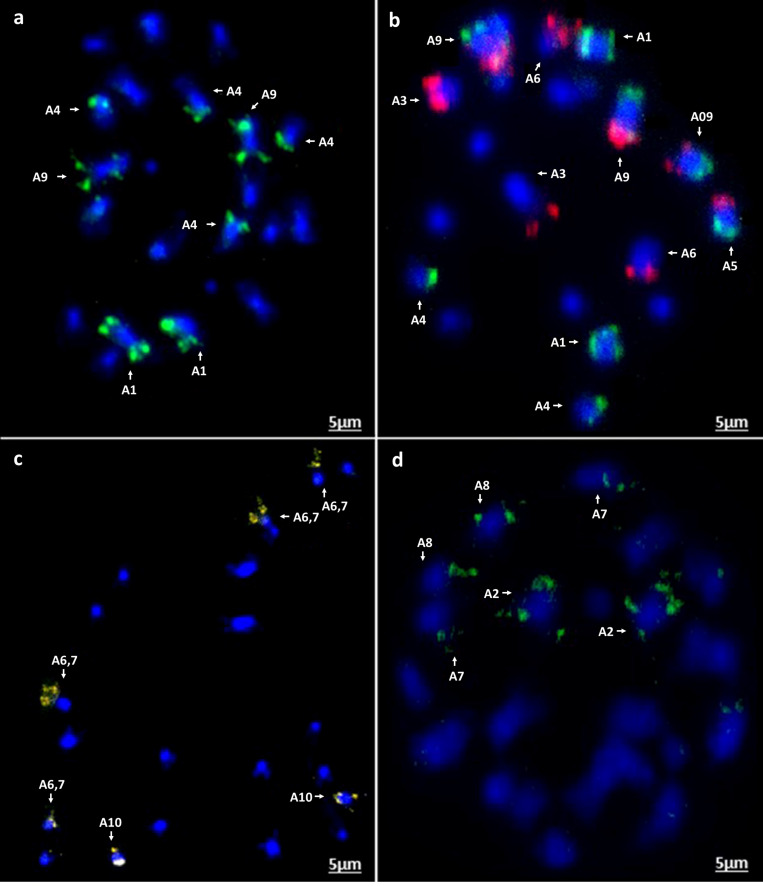
*In situ* hybridization on mitotic chromosome spreads of *B. rapa* cv. Chiffu-401 using four oligonucleotide libraries. Chromosomes are stained blue with DAPI and oligonucleotide probe hybridization sites are seen in the other colors **(a)** green-Atto488, **(b)** red-Atto550 and green-Atto488 as dual color *in situ*, **(c)** yellow-Atto594, and **(d)** green-Cy5. Scale bars = 5 μm.

**FIGURE 8:**
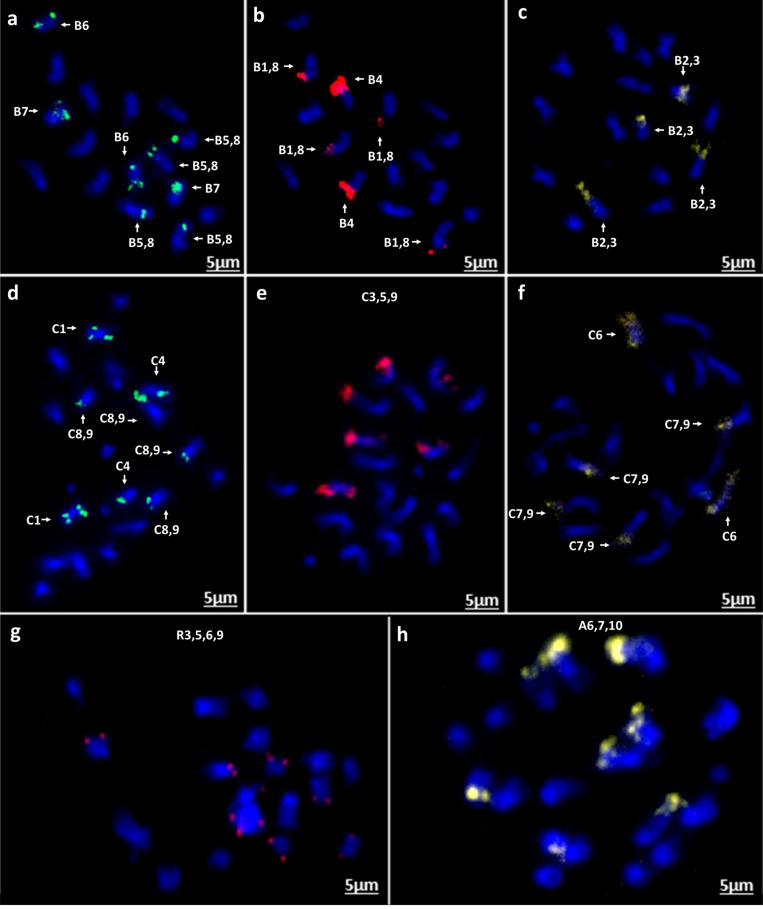
*In situ* hybridization on mitotic chromosome spreads of *B. nigra* using four oligonucleotide libraries as Figure 7. **(a)** green-Atto488, **(b)** red-Atto550, **(c)** yellow-Atto594; *B. oleracea* **(d)** green-Atto488, **(e)** red-Atto550 **(f)** yellow-Atto594; *R. sativus* **(g)** red-Atto550; **(h)** *B. rapa* (cv. Turnip Purple Top Milan) yellow-Atto594 demonstrating presence of an intraspecific chromosomal translocation compared to *B. rapa* cv. Chiffu-401 (Figure 7c).

Chromosomal *in situ* hybridization of the oligonucleotide probe pools on four diploid species *B. rapa* (cultivars Chiffu-401 and Purple Top Milan), *B. nigra*, *B. oleracea*, and *Raphanus sativus* validated the predicted outcomes. FISH signals derived from the four probes matched exactly to the patterns predicted in *B. rapa* Chiffu-401, *B. nigra*, and *R. sativus* (Figures 7a–d, 8a–h). With *B. oleracea*, one library (Atto 550, red) produced a pair of extra signals (Figure 8e). This might result from a regional duplication in the genotype used for validation of oligonucleotide probes, based on publicly available genome sequence of *B. oleracea*, or possible a small duplication that was not assembled. Notably, one commercial cultivar of *B. rapa* (turnip Purple Top Milan) exhibited a translocation with the yellow-colored library for Chiffu-401 (Figure 8h). Thus, these oligonucleotide libraries detect chromosomal translocations and duplications in different cultivars belonging to the same species.

## Discussion

We were able to exploit the reference genome of *B. rapa* to develop massive oligonucleotide pools from 18 chromosomal regions containing only single-copy sequences (Figure 1 and Table 1). These probe pools can be used to identify unambiguously all chromosomes and chromosome arms of crop brassica (A, B, and C) and radish (R) genomes in a single *in situ* hybridization experiment (Figures 7a–d, 8a–h). Our probes recognized corresponding homoeologous chromosomes and regions across these species and thus are able to anchor sequence maps, check sequence assemblies, and identify chromosomal rearrangements including translocations, inversions and fusion/fission events occurring both within and between species. Our probes designed to specific regions are specific, robust, and identify all chromosomes, unlike repetitive DNA probes developed using repetitive DNA sequences (e.g., 5S, 45S, Cent Br1 and Cent Br2, PBrSTR, PBnSTR, PBoSTR, PBnBH35, and pBcKB4; Harrison and Heslop-Harrison, 1995; Snowdon et al., 1997; Fukui et al., 1998; Kulak et al., 2002; Lim et al., 2005, 2007; He et al., 2015; Xu et al., 2016; Wang et al., 2017; Sun et al., 2019). The *in situ* hybridization mapping results are largely in concordance with physical genome sequence maps of the four species, achieved by high-coverage (deep) sequencing including use of long-molecule and mate-pair approaches. In the future, the high-quality reference genome sequence will be used to identify genetic polymorphisms including SNPs (single nucleotide polymorphisms) by re-sequencing, Genotyping-by-Sequencing (GBS) or RNA-seq approaches with relatively low coverage. However, chromosomal rearrangements are unlikely to be detected by either shallow or selective sequencing approaches. Using the oligonucleotide pools, we were able to detect an intra-specific rearrangement between the reference sequence of *B. rapa* Chiffu-401 (pak choi) and turnip Purple Top Milan (Figure 8h). The presence of such a translocation would restrict the ability to exchange genetic materials between the *B. rapa* varieties by making hybrids to exploit the diversity present. Our probes also detect chromosome rearrangements that precipitated differentiation of *Brassica* species from the ancestral crucifer karyotype. Oligonucleotide based karyotypes facilitate aligning and numbering of chromosomes in integration with the linkage maps or physical maps, and will also help to improve the quality of genome sequence assembly *via* identification of gaps or duplications in assembled plant genomes.

The strength and distribution of oligonucleotide signals varied between four species depending upon differences in genetic relationships and genomic sequences, and the results were robust between experiments. Short probes such as the oligonucleotides are valuable in combination with immunolabeling of chromosomal proteins in recombination or chromatin studies (Sepsi et al., 2018). As per phylogenetic reconstructions, A and C genomes of *Brassica* are more closely related to each other than to B and R genomes (Prakash et al., 2009). In our results, we also found that the C genome chromosomes generated the more similar signals to corresponding A genome chromosomes suggesting greater sequences or genetic similarity. More dispersed or dissimilar signals were obtained on the chromosomes of B and R genomes. The developed probes will also facilitate development of cytogenetic stocks, especially chromosome addition and substitution lines by identifying the chromosomes. Such chromosome stocks are eminently viable in brassicas as these crops can tolerate chromatin gain or loss because of the buffering provided by their palaeopolyploid nature (Cheng et al., 2014; Alix et al., 2017). Random chromosome addition (Prakash et al., 2009) or substitution lines developed in brassicas are stable (Banga, 1988; Gupta et al., 2016), and likely to be of increasing value for breeding as they allow characterization of genes of agronomic and quality relevance.

Oligonucleotide pools will be of significant value for identifying and tracing alien introgressions in brassicas, since the conservation of the low copy sequences, unlike many repetitive DNA elements, is relatively conserved between the sequenced crops and wild species. The genome sequences and gene diversity of many wild Brassicaceae genomes are being studied to expand the genepool available to breeders. The use of alien introgressions and the characterization of recombinant chromosomes is known to be of value in wheat, using probes that label whole alien chromosomes (Patokar et al., 2016), not available in brassicas. In some cases, BAC (Bacterial Artificial Chromosome) probes carrying the genes of interest for introgression in the Brassicaceae can be used (Niemela et al., 2012), but few new BAC libraries are being characterized or even maintained in the 21st century, and the hybridization is difficult to optimize. Because the oligonucleotides are designed in largely single-copy regions of the genome, there is a relatively high conservation with well-studied *Brassica* genomes. The computational pipelines as described in Figure 1 would be able to identify oligonucleotides with homology to regions assembled from low-coverage reads by mapping the wild genome reads to a phylogenetically distant reference genome, while excluding repetitive genome regions from the wild genomes, without having a high-quality assembly. Furthermore, the strategy would enable design of probes related to regions of interest, for example to track introgression of regions carrying genes of interest in a breeding program involving backcrossing.

Use of synthetic labeled oligonucleotides is convenient and efficient in ensuring all the probes are similarly labeled. However, it is relatively high-cost, requiring some 10 pmol of each probe, so for four labels costing more than United States $100 per slide. Amplification of the oligonucleotides *via* PCR makes the cost of probes, once synthesized, less prohibitive. Previous approaches have recommended oligonucleotide amplification *via* an RNA intermediate and extensive optimization of emulsion PCR steps which were not required with the two-step cycles and high denaturation temperature used here. We could show amplification of DNA probes *via* a robust PCR method to generate hundreds of ng of product from a few picomoles of oligonucleotide-pool. Theoretically, amplification can be selective and there may be self-priming products, but our results here showed no obvious differences using probes labeled during synthesis.

In conclusion, we could identify all *Brassica* chromosomes in the major A, B, and C genomes, with the use of massive pools of designed synthetic oligonucleotide probes. Following the design strategy including screening against new genome-wide, unbiased repetitive DNA sequence motifs, libraries can be made to target any appropriate chromosomal region. Appropriate designing of probes is critical as even a few repetitive motifs in the oligonucleotides, if not filtered out by the bioinformatic analysis can make the whole library less efficient.

## Data Availability Statement

The raw data supporting the conclusions of this article will be made available by the authors, without undue reservation.

## Author Contributions

JH-H, SB, and NA designed the experiments. NA performed the experiments. NA, MG, and JH-H performed the data analysis. NA, MG, SB, and JH-H wrote the manuscript. All authors approved the submission.

## Conflict of Interest

The authors declare that the research was conducted in the absence of any commercial or financial relationships that could be construed as a potential conflict of interest.

## Footnotes

1 http://brassicadb.org/brad/datasets/pub/Genomes/

## References

[B1] Alix K. Gérard P. R. Schwarzacher T. Heslop-Harrison J. S. (2017). Polyploidy and interspecific hybridization: partners for adaptation, speciation and evolution in plants. *Ann. Bot.* 120 183–194. 10.1093/aob/mcx079 28854567 PMC5737848

[B2] Amosova A. V. Bolsheva N. L. Zoshchuk S. A. Twardovska M. O. Yurkevich O. Y. Andreev I. O. (2017). Comparative molecular cytogenetic characterization of seven *Deschampsia* (Poaceae) species. *PLoS One* 12:e0175760. 10.1371/journal.pone.0175760 28407010 PMC5391082

[B3] Banga S. S. (1988). C-genome chromosome substitution lines in *Brassica juncea* (L.) Coss. *Genetica* 77 81–84. 10.1007/bf00057756 26913722

[B4] Beliveau B. J. Boettiger A. N. Avendaño M. S. Jungmann R. McCole R. B. Joyce E. F. (2015). Single-molecule super-resolution imaging of chromosomes and in situ haplotype visualization using Oligopaint FISH probes. *Nat. Commun.* 12 1–3. 10.1111/mmi.12942 25962338 PMC4430122

[B5] Bertioli D. J. Vidigal B. Nielen S. Ratnaparkhe M. B. Lee T. H. Leal-Bertioli S. C. (2013). The repetitive component of the A genome of peanut (*Arachis hypogaea*) and its role in remodelling intergenic sequence space since its evolutionary divergence from the B genome. *Ann. Bot.* 112 545–559. 10.1093/aob/mct128 23828319 PMC3718217

[B6] Braz G. T. He L. Zhao H. Zhang T. Semrau K. Rouillard J. M. (2018). Comparative oligo-FISH mapping: an efficient and powerful methodology to reveal karyotypic and chromosomal evolution. *Genetics* 208 513–523. 10.1534/genetics.117.300344 29242292 PMC5788518

[B7] Chalhoub B. Denoeud F. Liu S. Parkin I. A. Tang H. Wang X. (2014). Early allopolyploid evolution in the post-Neolithic *Brassica napus* oilseed genome. *Science* 345 950–953. 25146293 10.1126/science.1253435

[B8] Cheng F. Wu J. Fang L. Sun S. Liu B. Lin K. (2012). Biased gene fractionation and dominant gene expression among the subgenomes of *Brassica rapa*. *PLoS One* 7:e36442. 10.1371/journal.pone.0036442 22567157 PMC3342247

[B9] Cheng F. Wu J. Wang X. (2014). Genome triplication drove the diversification of Brassica plants. *Hortic. Res.* 15 14024–14030. 10.1038/hortres.2014.24 PMC4596316 26504539

[B10] Danilova T. V. Friebe B. Gill B. S. (2012). Single-copy gene fluorescence in situ hybridization and genome analysis: Acc-2 loci mark evolutionary chromosomal rearrangements in wheat. *Chromosoma* 121 597–611. 10.1007/s00412-012-0384-7 23052335

[B11] Fransz P. Armstrong S. Alonso-blanco C. Fischer T. C. Torres-Ruiz R. A. Jones G. (1998). Cytogenetics for the model system *Arabidopsis thaliana*. *Plant J.* 13 867–876. 9681023 10.1046/j.1365-313x.1998.00086.x

[B12] Fukui K. Nakayama S. Ohmido N. Yoshiaki H. Yamabe M. (1998). Quantitative karyotyping of three diploid *Brassica* species by imaging methods and localization of 45S rDNA loci on the identified chromosomes. *Theor. Appl. Genet.* 96 325–330. 10.1007/s001220050744 24710867

[B13] Gupta M. Mason A. S. Batley J. Bharti S. Banga S. Banga S. S. (2016). Molecular-cytogenetic characterization of C-genome chromosome substitution lines in *Brassica juncea* (L.) Czern and Coss. *Theor. Appl. Genet.* 129 1153–1166. 10.1007/s00122-016-2692-4 26913722

[B14] Han Y. Zhang T. Thammapichai P. Weng Y. Jiang J. (2015). Chromosome-specific painting in *Cucumis* species using bulked oligonucleotides. *Genetics* 200 771–779. 10.1534/genetics.115.177642 25971668 PMC4512542

[B15] Harrison G. E. Heslop-Harrison J. S. (1995). Centromeric repetitive DNA sequences in the genus *Brassica*. *Theor. Appl. Genet.* 90 157–165. 10.1007/bf00222197 24173886

[B16] He Q. Cai Z. Hu T. Liu H. Bao C. Mao W. (2015). Repetitive sequence analysis and karyotyping reveals centromere-associated DNA sequences in radish (*Raphanus sativus* L.). *BMC Plant Biol.* 15:105. 10.1186/s12870-015-0480-y 25928652 PMC4417506

[B17] Hou L. Xu M. Zhang T. Xu Z. Wang W. Zhang J. (2018). Chromosome painting and its applications in cultivated and wild rice. *BMC Plant Biol.* 18:110. 10.1186/s12870-018-1325-2 29879904 PMC5991451

[B18] Huang Y. Chen H. Han J. Zhang Y. Ma S. Yu G. (2020). Species-specific abundant retrotransposons elucidate the genomic composition of modern sugarcane cultivars. *Chromosoma* 129 45–55. 10.1007/s00412-019-00729-1 31848693

[B19] Idziak D. Betekhtin A. Wolny E. Lesniewska K. Wright J. Febrer M. (2011). Painting the chromosomes of Brachypodium-current status and future prospects. *Chromosoma* 120 469–479. 10.1007/s00412-011-0326-9 21667205 PMC3174371

[B20] Idziak D. Hazuka I. Poliwczak B. Wiszynska A. Wolny E. Hasterok R. (2014). Insight into the karyotype evolution of Brachypodium species using comparative chromosome barcoding. *PLoS One* 9:e93503. 10.1371/journal.pone.0093503 24675822 PMC3968144

[B21] Jiang J. Gill B. S. (2006). Current status and the future of fluorescence in situ hybridization (FISH) in plant genome research. *Genome* 49 1057–1068. 10.1139/g06-076 17110986

[B22] Kato A. Lamb J. C. Birchler J. A. (2004). Chromosome painting using repetitive DNA sequences as probes for somatic chromosome identi?cation in maize. *Proc. Natl. Acad. Sci. U.S.A.* 101 13554–13559. 10.1073/pnas.0403659101 15342909 PMC518793

[B23] Komuro S. Endo R. Shikata K. Kato A. (2013). Genomic and chromosomal distribution patterns of various repeated DNA sequences in wheat revealed by a fluorescence in situ hybridization procedure. *Genome* 56 131–137. 10.1139/gen-2013-0003 23659696

[B24] Koo D. H. Zhao H. Jiang J. (2016). Chromatin-associated transcripts of tandemly repetitive DNA sequences revealed by RNA-FISH. *Chromosome Res.* 24 467–480. 10.1007/s10577-016-9537-5 27590598

[B25] Krivankova A. Kopecky D. Stoces S. Dolezel J. Hribova E. (2017). Repetitive DNA: a versatile tool for karyotyping in *Festuca pratensis* huds. *Cyto Genome Res.* 151 96–105. 10.1159/000462915 28334706

[B26] Kulak S. Hasterok R. Maluszynska J. (2002). Karyotyping of *Brassica* amphidiploids using 5S and 25S rDNA as chromosome markers. *Hereditas* 137 79–80. 10.1034/j.1601-5223.2002.1360209.x 12369100

[B27] Kulikova O. Gualtieri G. Geurts R. Kim D. J. Cook D. Huguet T. (2001). Integration of the FISH pachytene and genetic maps of *Medicago truncatula*. *Plant J.* 27 49–58. 10.1046/j.1365-313x.2001.01057.x 11489182

[B28] Lagercrantz U. (1998). Comparative mapping between *Arabidopsis thaliana* and *Brassica nigra* indicates that *Brassica* genomes have evolved through extensive genome replication accompanied by chromosome fusions and frequent rearrangements. *Genetics* 150 1217–1228. 9799273 10.1093/genetics/150.3.1217 PMC1460378

[B29] Lagercrantz U. Lydiate D. J. (1996). Comparative genome mapping in *Brassica*. *Genetics* 144 1903–1910. 8978073 10.1093/genetics/144.4.1903 PMC1207737

[B30] Lan T. H. DelMonte T. A. Reischmann K. P. Hyman J. Kowalski S. P. McFerson J. (2000). An EST-enriched comparative map of *Brassica oleracea* and *Arabidopsis thaliana*. *Genome Res.* 10 776–788. 10.1101/gr.10.6.776 10854410 PMC310908

[B31] Lim K. B. De Jong H. Yang T. J. Park J. Y. Kwon S. J. Kim J. S. (2005). Characterization of rDNAs and tandem repeats in the heterochromatin of *Brassica rapa*. *Mol. Cell* 19 41–55. 15995362

[B32] Lim K. B. Yang T. J. Hwang Y. J. Kim J. S. Park J. Y. Kwon S. J. (2007). Characterization of the centromere and peri−centromere retrotransposons in *Brassica rapa* and their distribution in related *Brassica* species. *Plant J.* 49 173–183. 10.1111/j.1365-313x.2006.02952.x 17156411

[B33] Liu S. Liu Y. Yang X. Tong C. Edwards D. Parkin I. A. (2014). The Brassica oleracea genome reveals the asymmetrical evolution of polyploid genomes. *Nat. Commun.* 23:3930. 10.1038/ncomms4930 PMC4279128 24852848

[B34] Lyons E. Pedersen B. Kane J. Alma M. Ming R. Tang H. (2008). Finding and comparing syntenic regions among *Arabidopsis* and the outgroups papaya, poplar, and grape: CoGe with rosids. *Plant Physiol.* 148 1772–1781. 10.1104/pp.108.124867 18952863 PMC2593677

[B35] Lysak M. A. Berr A. Pecinka A. Schmidt R. McBreen K. Schubert I. (2006). Mechanisms of chromosome number reduction in *Arabidopsis thaliana* and related Brassicaceae species. *Proc. Natl. Acad. Sci. U.S.A.* 103 5224–5229. 10.1073/pnas.0510791103 16549785 PMC1458822

[B36] Lysak M. A. Cheung K. Kitschke M. Bureš P. (2007). Ancestral chromosomal blocks are triplicated in *Brassiceae* species with varying chromosome number and genome size. *Plant Physiol.* 145 402–410. 10.1104/pp.107.104380 17720758 PMC2048728

[B37] Lysak M. A. Koch M. A. Pecinka A. Schubert I. (2005). Chromosome triplication found across the tribe *Brassicae*. *Genome Res.* 15 516–525. 10.1101/gr.3531105 15781573 PMC1074366

[B38] Mandakova T. Lysak M. A. (2008). Chromosomal phylogeny and karyotype evolution in x= 7 crucifer species (Brassicaceae). *Plant Cell* 20 2559–2570. 10.1105/tpc.108.062166 18836039 PMC2590746

[B39] Mandakova T. Marhold K. Lysak M. A. (2013). The widespread crucifer species *Cardamine flexulosa* is an allotetraploid with a conserved subgenomic structure. *New Phytol.* 201 982–992. 10.1111/nph.12567 24400905

[B40] Mandakova T. Pouch M. Brock J. R. Al-Shehbaz I. A. Lysak M. A. (2019). Origin and evolution of diploid and allopolyploid camelina genomes were accompanied by chromosome shattering. *Plant Cell* 31 2596–2612. 31451448 10.1105/tpc.19.00366 PMC6881126

[B41] Meng Z. Zhang Z. Yan T. Lin Q. Wang Y. Huang W. (2018). Comprehensively characterizing the cytological features of Saccharum spontaneum by the development of a complete set of chromosome-specific oligo probes. *Front. Plant Sci.* 9:1624. 10.3389/fpls.2018.01624 PMC6232525 30459801

[B42] Mukai Y. Nakahara Y. Yamamoto M. (1993). Simultaneous discrimination of the three genomes in hexaploid wheat by multicolor ?uorescence in situ hybridization using total genomic and highly repeated DNA probes. *Genome* 36 489–494. 10.1139/g93-067 18470003

[B43] Niemela T. Seppänen M. Badakshi F. Rokka V. M. Heslop-Harrison J. P. (2012). Size and location of radish chromosome regions carrying the fertility restorer Rfk1 gene in spring turnip rape. *Chromosome Res.* 20 353–361. 10.1007/s10577-012-9280-5 22476396

[B44] Novak P. Neumann P. Pech J. Steinhaisl J. Macas J. (2013). RepeatExplorer: a galaxy-based web server for genome-wide characterization of eukaryotic repetitive elements from next-generation sequence reads. *Bioinformatics* 29 792–803. 10.1093/bioinformatics/btt054 23376349

[B45] Patokar C. Sepsi A. Schwarzacher T. Kishii M. Heslop-Harrison J. S. (2016). Molecular cytogenetic characterization of novel wheat-*Thinopyrum bessarabicum* recombinant lines carrying intercalary translocations. *Chromosoma* 125 163–172. 10.1007/s00412-015-0537-6 26238987

[B46] Pecinka A. Schubert V. Meister A. Kreth G. Klatte M. Lysak M. A. (2004). Chromosome territory arrangement and homologous pairing in nuclei of *Arabidopsis thaliana* are predominantly random except for NOR-bearing chromosomes. *Chromosoma* 113 258–269. 10.1007/s00412-004-0316-2 15480725

[B47] Perumal S. Koh C. S. Jin L. Buchwaldt M. Higgins E. Zheng C. (2020). High contiguity long read assembly of *Brassica nigra* allows localization of active centromeres and provides insights into the ancestral Brassica genome. *bioRxiv* 10.1101/2020.02.03.932665 PMC7419231 32782408

[B48] Peters S. A. Bargsten J. W. Szinay D. van de Belt J. Visser R. G. Bai Y. (2012). Structural homology in the Solanaceae: analysis of genomic regions in support of synteny studies in tomato, potato and pepper. *Plant J.* 71 602–614. 10.1111/j.1365-313x.2012.05012.x 22463056

[B49] Prakash S. Bhat S. R. Quiros C. F. Kirti P. B. Chopra V. L. (2009). Brassica and Its Close allies: cytogenetics and evolution. *Plant Breed. Rev.* 31 21–187. 10.1002/9780470593783.ch2

[B50] Qu M. Li K. Han Y. Chen L. Li Z. Han Y. (2017). Integrated karyotyping of woodland strawberry (*Fragaria vesca*) with oligopaint FISH probes. *Cytogenet. Genome Res.* 153 158–164. 10.1159/000485283 29262412

[B51] Said M. Hribova E. Danilova T. V. Karafiatova M. Cizkova J. Friebe B. (2018). The *Agropyron cristatum* karyotype, chromosome structure and cross-genome homoeology as revealed by fluorescence in situ hybridization with tandem repeats and wheat single-gene probes. *Theor. Appl. Genet.* 131 2213–2227. 10.1007/s00122-018-3148-9 30069594 PMC6154037

[B52] Salse J. Feuillet C. (2011). Palaeogenomics in cereals: modeling of ancestors for modern species improvement. *Comptes. Rendus. Biol.* 334 205–211. 10.1016/j.crvi.2010.12.014 21377615

[B53] Santos F. C. Guyot R. do Valle C. B. Chiari L. Techio V. H. Heslop-Harrison P. (2015). Chromosomal distribution and evolution of abundant retrotransposons in plants: gypsy elements in diploid and polyploid *Brachiaria forage* grasses. *Chromosome Res.* 23 571–582. 10.1007/s10577-015-9492-6 26386563

[B54] Schranz M. E. Lysak M. A. Mitchell-Olds T. (2006). The ABC’s of comparative genomics in the Brassicaceae: building blocks of crucifer genomes. *Trends Plant Sci.* 11 535–542. 10.1016/j.tplants.2006.09.002 17029932

[B55] Schubert I. Lysak M. A. (2011). Interpretation of karyotype evolution should consider chromosome structural constraints. *Trends Genet.* 27 207–216. 10.1016/j.tig.2011.03.004 21592609

[B56] Schwarzacher T. Heslop-Harrison P. (2000). *Practical in situ Hybridization.* Milton Park: BIOS Scientific Publishers, UK.

[B57] Sepsi A. Fábián A. Jäger K. Heslop-Harrison J. S. Schwarzacher T. (2018). ImmunoFISH: simultaneous visualisation of proteins and DNA sequences gives insight into meiotic processes in nuclei of grasses. *Front. Plant Sci.* 14:1193. 10.3389/fpls.2018.01193 PMC6102387 30154816

[B58] Šimoníková D. Nìmeèková A. Karafiátová M. Uwimana B. Swennen R. Doležel J. (2019). Chromosome painting facilitates anchoring reference genome sequence to chromosomes in situ and integrated karyotyping in banana (*Musa* spp.). *Front. Plant Sci.* 10:1503. 10.3389/fpls.2019.01503 PMC6879668 31824534

[B59] Snowdon R. J. Kohler W. Kohler A. (1997). Chromosomal localization and characterization of rDNA loci in the Brassica A and C genomes. *Genome* 40 582–587. 10.1139/g97-076 18464849

[B60] Song Z. Dai S. Bao T. Zuo Y. Xiang Q. Li J. (2020). Analysis of structural genomic diversity in *Aegilops umbellulata*, *Ae. markgrafii*, *Ae. comosa*, and *Ae. uniaristata* by fluorescence in situ hybridization karyotyping. *Front. Plant Sci.* 9:710. 10.3389/fpls.2020.00710 PMC7325912 32655588

[B61] Sun D. Wang C. Zhang X. Zhang W. Jiang H. Yao X. (2019). Draft genome sequence of cauliflower (*Brassica oleracea* L. var. botrytis) provides new insights into the C genome in *Brassica* species. *Horticulture Res.* 6:82. 10.1038/s41438-019-0164-0 PMC6804732 31645943

[B62] Szinay D. Wijnker E. van den Berg R. Visser R. G. de Jong H. Bai Y. (2012). Chromosome evolution in Solanum traced by cross−species BAC−FISH. *New Phytol.* 195 688–698. 10.1111/j.1469-8137.2012.04195.x 22686400

[B63] Tang H. Woodhouse M. R. Cheng F. Schnable J. C. Pedersen B. S. Conant G. (2012). Altered patterns of fractionation and exon deletions in *Brassica rapa* support a two-step model of paleohexaploidy. *Genetics* 190 1563–1574. 10.1534/genetics.111.137349 22308264 PMC3316664

[B64] Tang X. Szinay D. Lang C. (2008). Cross-species bacterial artificial chromosome-fluorescence in situ hybridization painting of the tomato and potato chromosome 6 reveals undescribed chromosomal rearrangements. *Genetics* 180 1319–1328. 10.1534/genetics.108.093211 18791231 PMC2581937

[B65] Wang G. X. He Q. Y. Macas J. Novak P. Neumann P. Meng D. X. (2017). Karyotypes and distribution of tandem repeat sequences in *Brassica nigra* determined by fluorescence in situ hybridization. *Cytogenet. Genome Res.* 152 158–165. 10.1159/000479179 28810257

[B66] Wang J. Lydiate D. J. Parkin I. A. Falentin C. Delourme R. Carion P. W. (2011). Integration of linkage maps for the amphidiploid *Brassica napus* and comparative mapping with *Arabidopsis* and *Brassica rapa*. *BMC Genomics* 12:101. 10.1186/1471-2164-12-101 PMC3042011 21306613

[B67] Wang W. Huang S. Liu Y. Fang Z. Yang L. Hua W. (2012). Construction and analysis of a high-density genetic linkage map in cabbage (*Brassica oleracea* L. var. capitata). *BMC Genomics* 13:523. 10.1186/1471-2164-13-523 23033896 PMC3542169

[B68] Xin H. Zhang T. Han Y. Wu Y. Shi J. Xi M. (2018). Chromosome painting and comparative physical mapping of the sex chromosomes in *Populus tomentosa* and *Populus deltoides*. *Chromosoma* 127 313–321. 10.1007/s00412-018-0664-y 29520650

[B69] Xiong Z. Pires J. C. (2011). Karyotype and identification of all homoeologous chromosomes of allopolyploid *Brassica napus* and its diploid progenitors. *Genet* 187 37–49. 10.1534/genetics.110.122473 21041557 PMC3018299

[B70] Xu Z. Xie B. Wu T. Xin X. Man L. Tan G. (2016). Karyotyping and identifying all of the chromosomes of allopolyploid *Brassica juncea* using multicolor FISH. *Crop J.* 4 266–274. 10.1016/j.cj.2016.05.006

[B71] Yang J. Liu D. Wang X. Ji C. Cheng F. Liu B. (2016). The genome sequence of allopolyploid *Brassica juncea* and analysis of differential homoeolog gene expression influencing selection. *Nat. Genet.* 48 1225–1232. 10.1038/ng.3657 27595476

[B72] Zhang X. Scheuring C. Tripathy S. Xu Z. Wu C. Ko A. (2006). An integrated BAC and genome sequence physical map of *Phytophthora sojae*. *Mol. Plant Microbe Interact.* 19 1302–1310. 10.1094/mpmi-19-1302 17153914

[B73] Zhang Y. Zheng C. Sankoff D. (2018). Pinning down ploidy in paleopolyploid plants. *BMC Genomics* 19:287. 10.1186/s12864-018-4624-y 29745846 PMC5998896

